# Reconciling corruption with conservation triage: Should investments shift from the last best places?

**DOI:** 10.1371/journal.pbio.2005620

**Published:** 2018-08-31

**Authors:** Craig Packer, Stephen Polasky

**Affiliations:** 1 Department of Ecology, Evolution, and Behavior, University of Minnesota, St. Paul, Minnesota, United States of America; 2 School of Life Sciences, University of KwaZulu-Natal, Westville, South Africa; 3 Department of Applied Economics, University of Minnesota, St. Paul, Minnesota, United States of America

## Abstract

Considerable outside funding will be required to overcome the financial shortfalls faced by most of Africa’s protected areas. Given limited levels of external support, it will be essential to allocate these funds wisely. While most recent studies on conservation triage have recommended prioritizing reserves with the highest remaining conservation value (the “last best places”), such investments are complicated by the fact that these same reserves often attract the greatest revenues from ecotourism and thus the most attention from corrupt local governments. Alternatively, philanthropic organizations might achieve greater returns from investing in the management of neglected areas with lower current conservation value but with less financial leakage from corruption. We outline here how high levels of corruption could favor a strategy that shifts investments away from the last best places.

Funding for protected areas is well below recommended levels worldwide [1]. Underfunding directly translates to deteriorating protected-area status and a concomitant decline in biodiversity. The problem is exemplified by the current conservation crisis in Africa. Across the entire continent, black rhinos have largely disappeared [2], elephant numbers have dropped 30% over the past decade [3], giraffes have declined by 40% in the past 30 years [2], and cheetah numbers have shrunk by 20% over the past 20 years [4]; across West, Central, and East Africa, lion populations have declined by nearly 50% over the past 24 years [5]. Conservation challenges intensify with increasing human population densities, and human density in sub-Saharan Africa is expected to match the present-day density of India by 2060 [6]. While protected areas in North America and Western Europe are mostly funded by tax revenues, Africa is home to many of the poorest countries on earth [7], with little tax revenue and pressing social needs, leaving little public funding available for conservation. Thus, wildlife has been expected “to pay its own way.” However, photo-tourism only raises significant revenues in a few high-profile areas, and sport hunting is only viable in areas that support adequate populations of trophy species. Most African reserves cannot in fact “pay their own way.”

Given the shortfall in locally generated funding and the rapid decline of remaining wildlife populations, there is an urgent need for international subsidies [8]. Realistically, though, such funding is only likely to fill a small fraction of the revenue gap. Thus, it is essential to allocate limited conservation funds where they will have the greatest conservation impact. Most thinking about conservation “triage” focuses on directing resources to conservation reserves with the greatest return on investment [9–13]. Conservation areas vary in current and potential species richness, wildlife abundance, intactness, ecosystem functions, and ecosystem services. Thus, conservation agencies generally consider some combination of these features, which we refer to collectively as “conservation value,” giving priority to reserves with high conservation value (“the last best places”) while avoiding wasting resources on what appear to be sites with low conservation value [14].

Conservation investment in a nation’s wildlife sector is further complicated by inefficient government bureaucracy and corruption [15,16], which may reduce the effectiveness of conservation investments funneled through governmental agencies. In this paper, we outline a simple thought experiment illustrating how corruption might affect conservation strategies. Though we focus on Africa, the following arguments apply to other regions with pressing conservation needs and corrupt or inefficient governments, such as Southeast Asia and Latin America [17–19].

In 2016, countries in sub-Saharan Africa comprised 17 of 30 of the most corrupt and zero of 30 of the least corrupt governments in Transparency International’s Corruption Perceptions Index [20]. With corruption, resources intended for conservation can be diverted to enrich well-connected individuals, ensure the re-election of the ruling political party, or allow government agencies to fund other activities besides wildlife management. Corruption can also take the form of payments to allow poaching or illegal resource harvests within reserves [19]. Taking corruption and the likely diversion of conservation investments into account could make investments in what initially appear to be lower value conservation sites more attractive than focusing solely on funding the “last best places.”

Although there may be methods for minimizing corruption in government-run reserves (see below), we seek here to show the potential value of selectively restoring currently low-conservation value wildlife reserves that have essentially been abandoned by government authorities. In recent years, several philanthropic organizations have negotiated long-term contracts with African governments to gain exclusive management rights to protected areas that had previously generated little or no revenue; thus, the national governments have suffered virtually no opportunity costs by ceding control to outside agencies. After securing management rights for the publicly owned reserve, the ecophilanthropists secured the perimeters, reintroduced lost species, and restored biodiversity in the area. For example, African Parks (AP) is a conservation nongovernmental organization (NGO) that manages 13 national parks in 9 African countries and recently returned lions to Malawi and Rwanda and restored rhinos to Rwanda, and the AP reserve in Chad is home to the fastest growing elephant population in Africa. This success, however, required significant investment. AP reported expenditures of US$34.8 million with only approximately US$3 million in park revenues in 2016; 90% of costs were covered by international donors [21]. Similarly, ecophilanthropists such as Greg Carr and Paul Tudor Jones have made extensive personal expenditures to restore parks in Mozambique [22], Tanzania, Zambia, and Zimbabwe.

Conservation investments will typically yield different returns across reserves because of differing conservation values and implementation costs. However, corruption can also influence conservation returns, and levels of corruption may often vary with reserve quality. Sites that attract the most tourist revenues are the most tempting targets for control by corrupt officials. Thus, it is noteworthy that few of the recently restored sites mentioned above would have been considered prime tourist destinations or biodiversity hotspots 10–15 years ago.

We consider the potential impact of corruption on conservation priorities using a simple thought experiment. We contrast the difference between reserves with high conservation value that also generate high tourism returns and concomitant levels of corruption versus reserves with lower conservation value, little tourism revenue and therefore little government interest. In our simple approach, the cost of maintaining a reserve at its full conservation value is US$100,000 per year, and the overall donor budget is US$200,000 per year. We compare conservation outcomes under two strategies. The first strategy seeks to fully meet the funding shortfalls of the top four biodiversity reserves (“last best places”) shown in Fig 1A; note that US$200,000 is sufficient to meet the funding shortfall of all four of these sites. As shown in Fig 1B, these reserves have the highest potential conservation value because they have the highest conservation value when fully conserved; they also have the highest current conservation value because they are already partially conserved. The second strategy invests in the full restoration of two sites with lower potential conservation value; they also have lower current conservation value because of government neglect. Spending the entire budget on these two sites similarly exhausts the entire US$200,000 budget.

**Fig 1:**
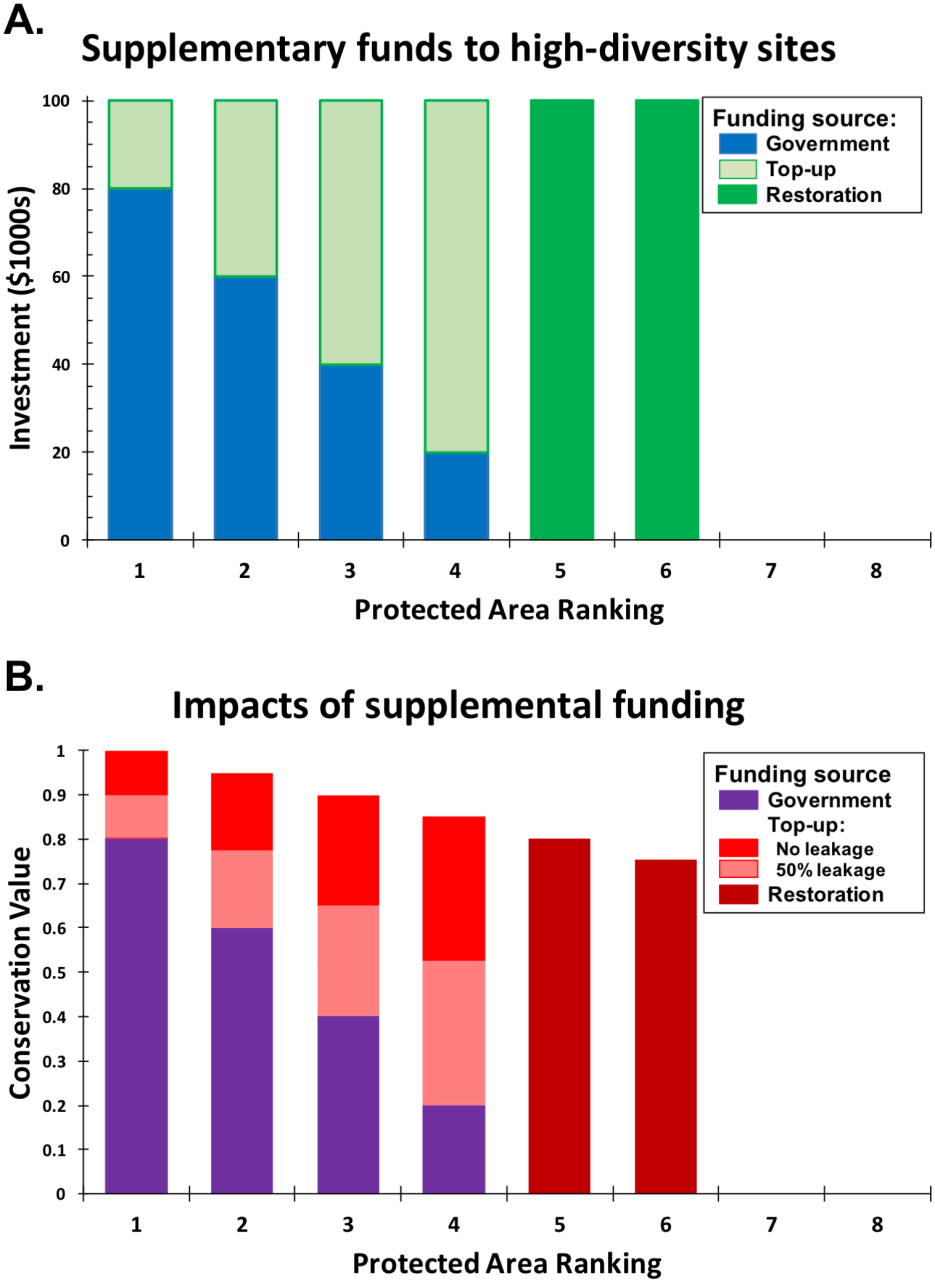
Alternative funding strategies and associated outcomes, assuming management costs of US$100,000 to reach potential biodiversity value for each site but with declining potential biodiversity value across sites. Sites are ordered in terms of decreasing potential biodiversity value. A. Governmental funding declines with decreasing biodiversity value of the site (blue), and conservation agencies make up the shortfalls either as top-ups for the four “last best places” (pale green for sites 1–4) or as the full restoration costs of the two lowest ranking sites (dark green for sites 5–6). B. The realized biodiversity value at each site is equal to the full potential value multiplied by the fraction of the current government budget relative to the necessary funding (purple). The impact of additional investment from external supplements depends on the degree of corruption at that site. In the absence of corruption, top-up supplements to the four best sites increase biodiversity values to their maximum (solid red). If half of the funds are diverted by corruption, inputs have only half the impact (pink). In the absence of governmental interference, restoration funding bolsters biodiversity to its full potential (brown).

In the absence of corruption, the last-best-places strategy enables the top four reserves to regain their full conservation value, resulting in conservation gains shown by the red bars in Fig 1B and generating a greater overall gain than investment in the two low-conservation value sites (brown bars in Fig 1B). However, suppose that the four “best” sites are subject to corruption while the neglected sites are not. Using Public Expenditure Tracking Surveys, leakage of donor aid in Africa’s healthcare sector is known to range from 40%–80% [23]. The pink bars in Fig 1B thus illustrate conservation gains if investment in the top four reserves are subject to 50% losses from corruption. In contrast, low-ranking areas do not generate revenue, thus donors are not subjected to government interference, and external funding fully restores the reserves to their potential conservation value (again indicated by the brown bars in Fig 1B). In this case, the increase in conservation value from investing in the two low-ranking areas generates a higher conservation return than investments in the top four reserves that are all subject to corruption. Thus, with sufficient levels of corruption, investment priorities should switch from “the last best places” to lower-ranked reserves that are not subject to corruption, a decision that ultimately arises because governments do not consider lesser areas capable of generating sufficient revenue to justify state-run management.

Our thought experiment suggests that, where corruption is tightly linked to sites with the greatest existing revenue, conservation priorities should shift toward lower conservation value reserves and away from the last best places. However, Fig 1 is merely illustrative, and since corruption varies widely across countries, this shift will not hold everywhere. Further, even where corruption is endemic, increasing the transparency and accountability for how funds are spent, investing in on-site presence to increase monitoring, and tying future funding to performance can all potentially reduce corruption and increase the effectiveness of conservation investment [24].

Fig 1 also takes the rate of corruption as exogenous and fixed. In reality, of course, corruption is variable and changes with conditions. So for example, even though current lower ranked sites do not attract corruption because there is no revenue associated with the reserve, successful management will eventually make the reserves attractive targets for corrupt government officials, who may then attempt to renationalize them to skim off the profits. A challenge for future investment is whether conservation entities will be allowed to keep or renew their leases once they have restored wildlife and made reserves attractive to tourism. Post-investment renegotiations are thus vulnerable to “the hold-up problem” [25, 26]. To guard against this threat, management contracts should be sufficiently long and sufficiently secure to make the initial investment worthwhile. Another option for avoiding the hold-up problem is to make continued investments in the parks (and associated profit-sharing with range-state governments) contingent on further cooperation from the government, so that officials are not tempted to “kill the goose that lays the golden egg.”

## Abbreviations

AP: African Parks
NGO: nongovernmental organization
